# Phylogenetic Curved Optimal Regression for Adaptive Trait Evolution

**DOI:** 10.3390/e23020218

**Published:** 2021-02-10

**Authors:** Dwueng-Chwuan Jhwueng, Chih-Ping Wang

**Affiliations:** Department of Statistics, Feng-Chia University, Taichung 40724, Taiwan; a09760381100@gmail.com

**Keywords:** adaptive trait evolution, approximate Bayesian computation, geometric Brownian motion, geometric Ornstein–Uhlenbeck process, phylogenetic comparative analysis

## Abstract

Regression analysis using line equations has been broadly applied in studying the evolutionary relationship between the response trait and its covariates. However, the characteristics among closely related species in nature present abundant diversities where the nonlinear relationship between traits have been frequently observed. By treating the evolution of quantitative traits along a phylogenetic tree as a set of continuous stochastic variables, statistical models for describing the dynamics of the optimum of the response trait and its covariates are built herein. Analytical representations for the response trait variables, as well as their optima among a group of related species, are derived. Due to the models’ lack of tractable likelihood, a procedure that implements the Approximate Bayesian Computation (ABC) technique is applied for statistical inference. Simulation results show that the new models perform well where the posterior means of the parameters are close to the true parameters. Empirical analysis supports the new models when analyzing the trait relationship among kangaroo species.

## 1. Introduction

Species evolve across generations. For quantitative-trait evolution, scientists apply phylogenetic comparative methods (PCMs) to study the evolutionary relationship of a group of related species where a phylogenetic tree is incorporated for describing affinity among species [1,2,3,4,5,6,7,8]. Most current regression models in PCMs assume that the response trait variable *y* is linear with its covariates *x*s where the estimated line equation (e.g., $y=b_{0}+\sum_{i=1}^{n}b_{i}x_{i}$) is used to predict the response trait [8,9,10]. However, the allometric relationship between body mass and other organisms is also often observed in nonlinear form (i.e., $y=kx^{a}$). Logarithm transformation (logy=logk+alogx) is usually considered as a regular procedure prior to analysis [11]. From a statistical perspective, log transformation on the data reduces skewness, decreasing the variability, conforming data close to the normal distribution, and placing dependent variable and covariates in a linear-like relationship [12,13,14]. From an evolutionary perspective, because most traits of particular species fall within a certain range, interpreting trait changes using raw scales may produce unreasonable results. Hence, convex transformation by the logarithm function is often applied to convert the raw data of the interval type into the ratio type. This has particular advantages, for example, a change in body mass of 0.2 kg might not be important for a male red kangaroo with a weight from 55 to 90 kg, but probably matters substantially for a wallaby with a weight of about 1.6 kg; a 1.36% change in body mass for both species is interpretable under log-transformed data.

**Figure 1:**
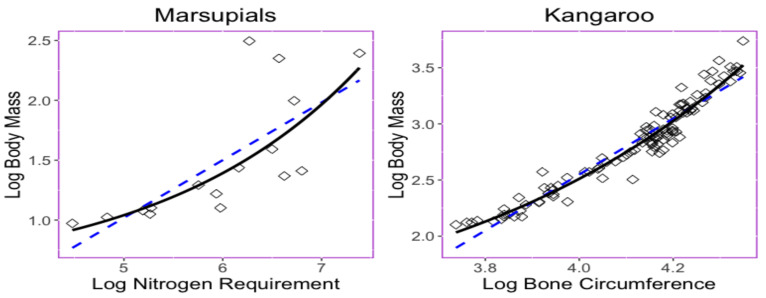
Scatter plots and the relationship of the bivariate trait dataset described by the least squares regression lines or curves. (**left**) Relationship between maintenance nitrogen requirement and body mass in marsupials [16,17]. Exponential curve y=0.486+0.047exp(0.490x) (RMSD =0.341) and line equation y=−1.389+0.482x (RMSD =0.344) shown. (**right**) Relationship between bone circumference and the body mass in kangaroos [18]. Exponential curve y=1.051+0.003exp(1.510x) (RMSD =0.092) and line y=−7.407+2.489x (RMSD =0.112) shown.

Nevertheless, even the log transformation helps to convert the trait relationship from nonlinear into a moderate linear type, and there exists a nonlinear relationship among some log-transformed data [15]. The trait relationship shown in Figure 1 provides two examples in which nonlinear exponential regressions could provide a better fit with less predicted errors than those obtained when using linear regression. The left panel in Figure 1 displays the bivariate relationship between the body mass (*x*) and the maintenance nitrogen requirement (*y*) in the log scale of the marsupial species [16,17]. The exponential equation y=0.486+0.047exp(0.490x) has a root mean square deviation with a value of 0.341, while the linear regression model y=−1.389+0.482x has a root mean square deviation 0.344.

The right panel in Figure 1 displays relationship between the thigh-bone (femoral) circumference (*x*) and body mass (*y*) of the kangaroo species [18] is shown Figure 1. The exponential equation y=1.051+0.003exp(1.510x) has a root mean square deviation with a value of 0.092, while the linear regression model y=−7.407+2.489x has a root mean square deviation of 0.112. Parameters $\beta_{1},\beta_{2},\beta_{3}$ in the exponential curve $y=\beta_{1}+\beta_{2}\exp\left(\beta_{3}x\right)$ were estimated under a least-squares method performed using the following step. Since $\beta_{1}$ adds to the complexity of the model, an estimate of $\beta_{1}$ is established by using the half value of the minimum of the responses; then, traits are subtracted from this value, which yields the model $y-\beta_{1}=\beta_{2}\exp\left(\beta_{3}x\right)$. Parameters $\beta_{2}$ and $\beta_{3}$ are estimated through the least-squares method for the model $\log\left(y-\beta_{1}\right)=\log\left(\beta_{2}\right)+\beta_{3}x$.

In the marsupial and kangaroo datasets, exponential regression models yield to smaller RMSD than those in linear regression models. Conceiving that the potential use of exponential regression models to study phylogenetic-traits relationships, the empirical datasets in Figure 1 call for novel phylogenetic comparative methods. In this framework, we developed models for adaptive trait evolution where the optimum of the trait undergoes stabilizing selection and has an exponential relationship with the predictor trait. Our work is distinguished from the work in [19], which mainly makes use of multiple linear regression. Our ultimate goal was to provide feasible models for scientists to analyze their valuable data for research.

Prior to developing new models, the background of phylogenetic adaptive trait evolution was introduced follows. Hansen et al. [20] developed a popular model (OUBM model) for phylogenetic adaptive trait evolution where the response trait variable is assumed following an Ornstein–Uhlenbeck (OU) process dynamic where the optimum of the response trait is assumed with a linear relationship with Brownian motion (BM) covariates. Later, various scientists made further efforts to expand the OUBM model of Hansen et al. via considering an Ornstein–Uhlenbeck process covariates (OUOU model) [21,22], a Cox–Ingersoll–Ross process for rate evolution [19], or extending the OUBM model to the multivariate case [23,24,25].

In general, the generalized model for phylogenetic adaptive trait evolution assumes that trait variable $y_{t}$ solves stochastic differential equation (SDE) in Equation (1):

(1) $$dy_{t}=\alpha_{t}^{y}\left(\theta_{t}^{y}-y_{t}\right)dt+\sigma_{t}^{y}dW_{t}^{y},$$

where parameter $\alpha_{t}^{y}$ is the force that pulled the trait back to its optimum $\theta_{t}^{y}$, parameter $\sigma_{t}^{y}$ is called the evolutionary rate for the trait variable $y_{t}$, and $W_{t}^{y}$ is a Wiener process with independent Gaussian increment, with mean 0 and variance *t*. Let $\alpha_{t}^{y}=\alpha_{y}$ and $\sigma_{t}^{y}=\sigma_{y}$ be constants. By multiplying the integrating factor $\exp(\alpha_{y}t)$ and then integrating on both sides of Equation (1), $y_{t}$ can be expressed explicitly, as shown in Equation (2)

(2) $$\begin{array}{c} y_{t}=\exp\left(-\alpha_{y}t\right)y_{0}+\int_{0}^{t}\alpha_{y}\exp\left(-\left(\alpha_{y}t-\alpha_{y}s\right)\right)\theta_{s}^{y}ds+\sigma_{y}\int_{0}^{t}\exp\left(-\left(\alpha_{y}t-\alpha_{y}s\right)\right)dW_{s}^{y}, \end{array}$$

where $\exp\left(-\alpha_{y}t\right)y_{0}$ is a deterministic term with initial condition $y_{0}$ at t=0, and term $\sigma_{y}\int_{0}^{t}\exp\left(-\left(\alpha_{y}t-\alpha_{y}s\right)\right)dW_{s}^{y}$ is a stochastic integral with respect to $W_{s}^{y}$, and is, again, a Gaussian variable with mean 0 and variance $\sigma_{y}^{2}\left(1-\exp\left(-2\alpha_{y}t\right)\right)/\left(2\alpha_{y}\right)$ (obtained by applying Itô isometry [26]) and

(3) $$Ⓐ=\exp\left(-\alpha_{y}t\right)\int_{0}^{t}\alpha_{y}\exp\left(\alpha_{y}s\right)\theta_{s}^{y}ds$$

is an integral with respect to time.

Optimal $\theta_{t}^{y}$, has a functional relationship with the covariate $x_{t}$ represented in Equation (4)

(4) $$\theta_{t}^{y}=f\left(\beta ,x_{t}\right),$$

where β is the vector of regression parameters.

In Equation (4), when optimum $\theta_{t}^{y}$ and covariate trait variable $x_{t}$ are in a linear relationship (i.e., $\theta_{t}^{y}=\beta_{0}+\sum_{i=1}^{p}\beta_{i}x_{i,t}$ where $x_{i,t},i=1,\cdots p$ are identical independently distributed continuous stochastic random variables), the dynamics of $\theta_{t}^{y}$ can be characterized through identifying the dynamics of the linear combination of identical independent distributed covariates $x_{i,t},i=1,2,\cdots ,p$. For Gaussian process covariates $x_{t}$s, optimal $\theta_{t}^{y}$ follows a Brownian motion if covariates $x_{t}$s follows Brownian motion (i.e., $dx_{i,t}=\sigma_{x_{i}}dW_{t}^{x_{i}}$), called the OUBM model [20]. On the other hand, $\theta_{t}^{y}$ is an OU process if $x_{i,t}$s are OU processes (i.e., $dx_{i,t}=\alpha_{x_{i}}\left(\theta_{x_{i}}-x_{i,t}\right)dt+\sigma_{x_{i}}dW_{t}^{x_{i}}$), called the OUOU model [21].

In this work, we assumed that an exponential relationship existed between trait optimum $\theta_{t}^{y}$ and its covariate $x_{t}$. The development of the new models is described as follows. When assuming an exponential relationship between the optimum $\theta_{t}^{y}$ and a Brownian motion covariate $x_{t}$, the optimum $\theta_{t}^{y}$ follows a well known geometric Brownian motion [27]. By assuming an exponential relationship between optimum $\theta_{t}^{y}$ and its Ornstein–Uhlenbeck process-type covariate $x_{t}$, the optimum $\theta_{t}^{y}$ follows a geometric Ornstein–Uhlenbeck process [28].

We assumed that the covariate trait variable $x_{t}$ evolved under Gaussian processes (e.g., Brownian motion or Ornstein–Uhlenbeck process); hence, the analytic expression of Ⓐ in Equation (3) depends on the expression between $\theta_{t}^{y}$ and its covariate $x_{t}$. Both evolutionary rate ($\sigma_{t}^{y}$) and force $\alpha_{t}^{y}$ in Equation (1) are assumed to be positive constants throughout this work (i.e., $\alpha_{t}^{y}=\alpha_{y}>0,\sigma_{t}^{y}=\sigma_{y}>0$). Hence, we focused on developing of models by implementing the curved relationship between optimum $\theta_{t}^{y}$ and its covariate $x_{t}$. The new model is named OUGBM (see Section 2.1.1) when trait $y_{t}$ represented in Equation (2) admits a generalized OU process dynamic, and its optimum $\theta_{t}^{y}$ has an exponential relationship with Brownian motion covariates $x_{t}$. The new model is named OUGOU (see Section 2.1.2) if $y_{t}$ admits a generalized OU process, and $\theta_{t}^{y}$ has an exponential relationship with OU process covariates $x_{t}$. We also implemented the OUBM (see Section 2.2.1) and OUOU (see Section 2.2.2) models for comparison with the new models. Since species are evolutionarily related, the models were developed with the assumption that evolutionary dependency among a group of species is along a given root phylogenetic tree (see Section 2.3). Due to those new models’ lack of model likelihood, we propose the use of the approximate Bayesian computation procedure for model inference (see Section 2.4).

## 2. Materials and Methods

### 2.1. Optimal Exponential Regression

Consider an exponential relationship between the optimum and its covariate as follows

(5) $$\theta_{t}^{y}=f\left(\beta ,x_{t}\right)=\beta_{1}+\beta_{2}\exp\left(\beta_{3}x_{t}\right).$$

The relationship in Equation (5) is commonly applied in growth/decay studies with $\beta_{1}$ representing the value of maximal growth (if $\beta_{3}<0$) or minimal decay (if $\beta_{3}>0$). By using Equation (5), two models (OUGBM and OUGOU) were developed, as reported in Section 2.1.1 and Section 2.1.2, respectively.

#### 2.1.1. OUGBM Model

Let $x_{t}$ be a Brownian motion random variable that solves the SDE $dx_{t}=\sigma_{x}dW_{t}^{x}$ (i.e., $\mu_{t}=0$ and $\sigma_{t}=\sigma_{x}$ in the SDE $dx_{t}=\mu_{t}dt+\sigma_{t}dW_{t}^{x}$. Suppose the optimum of the response trait $\theta_{t}^{y}$ has an exponential relationship with $x_{t}$, as shown in Equation (5). The first step is to express $\theta_{t}^{y}$ in terms of model parameters $\sigma_{x}$ in $x_{t}$. By taking a derivative in Equation (5) with respect to *t*, one has $d\theta_{t}^{y}=\beta_{2}d\exp\left(\beta_{3}x_{t}\right)$. Let $f\left(t,x\right)=\exp\left(\beta_{3}x\right)$ with partial derivative $f_{t}=0,f_{x}=\beta_{3}\exp\left(\beta_{3}x\right)$ and $f_{xx}=\beta_{3}^{2}\exp\left(\beta_{3}x\right)$. By applying Itô’s lemma [26] $df=\left(f_{t}+\mu_{t}f_{x}+\sigma_{t}^{2}f_{xx}/2\right)dt+\sigma_{t}f_{x}dW_{t}$, one has

$$d\exp\left(\beta_{3}x_{t}\right)=\left(\sigma_{x}^{2}\beta_{3}^{2}\exp\left(\beta_{3}x_{t}\right)/2\right)dt+\left(\sigma_{x}\beta_{3}\exp\left(\beta_{3}x_{t}\right)\right)dW_{t},$$

which is known as the SDE for a geometric Brownian motion random variable $f(x_{t})$ with constant of percentage drift parameter $\mu =\sigma_{x}^{2}\beta_{3}^{2}/2$ and a constant of percentage volatility parameter $\sigma =\sigma_{x}\beta_{3}$. The analytical solution is

$$f\left(x_{t}\right)=f\left(x_{0}\right)\exp\left(\left(\mu -\sigma^{2}/2\right)+\sigma_{x}W_{t}\right).$$

Plugging $f\left(x_{t}\right)=\exp\left(\beta_{t}x_{t}\right)$ into Equation (5) and then simplifying the equation yields to an explicit representation for the optimum as follows.

(6) $$\theta_{t}^{y}=\beta_{1}+\beta_{2}\exp\left(\beta_{3}\left(x_{0}+\sigma_{x}W_{t}^{x}\right)\right).$$

To draw a sample for trait $y_{t}$ considering the expression of $y_{t}$ in Equation (2), it suffices to recognize the dynamics of Ⓐ in Equation (3), where $Ⓐ=\int_{0}^{t}\alpha_{y}\exp\left(-\alpha_{y}\left(t-s\right)\right)\theta_{s}^{y}ds$. This can be performed by replacing $\theta_{t}^{y}$ with $\beta_{1}+\beta_{2}\exp\left(\beta_{3}\left(x_{0}+\sigma_{x}W_{t}^{x}\right)\right)$ in Equation (6), which yields to

$$Ⓐ=\beta_{1}\left(1-\exp\left(-\alpha_{y}t\right)\right)+\beta_{2}\alpha_{y}\exp\left(-\alpha_{y}t+\beta_{3}x_{0}\right)\int_{0}^{t}\exp\left(\alpha_{y}s+\beta_{3}\sigma_{x}W_{s}^{x}\right)ds,$$

where $\int_{0}^{t}\exp\left(\alpha_{y}s+\beta_{3}\sigma_{x}W_{s}^{x}\right)ds:=S_{t}$ is a definite integral with respect to time, and the integrand $\exp(\alpha_{y}s+\beta_{3}\sigma_{x}W_{s}^{x})$ is a geometric Brownian motion variable [29,30,31].

Currently there is no analytical expression for $S_{t}$. The authors in [31,32] extensively studied the problem and provided a numerical solution through the Laplace transform. In particular, when *t* approaches to *∞*, and the reciprocal of $S_{t}$ has a limit distribution of gamma type with shape parameter $\left(2\alpha_{y}\right)/\left(\beta_{3}^{2}\sigma_{x}^{2}\right)$ and scale parameter $(\beta_{3}^{2}\sigma_{x}^{2})/2$ for $\alpha_{y}>0$ at t→∞ (see Prop. 4.4.4 in [30]). In our modeling framework, since *t* represented evolutionary time and was of finite value (i.e., 0<t<1 after scaling tree in the models), samples of $S_{t}$ were drawn from the definite integral of a geometric Brownian motion variable $\exp(\alpha_{y}t+\beta_{3}\sigma_{x}W_{t}^{x})$ with respect to time on time domain [0,t] using Simpson’s rule [33]. Hence, given $t,\alpha_{y},\sigma_{x},\sigma_{y}$, samples of trait variables $y_{t},x_{t}$ and $\theta_{t}^{y}$ were accordingly drawn with the aid of R package pracma [34] to compute the stochastic integral.

#### 2.1.2. OUGOU Model

Let $x_{t}$ be the Ornstein–Uhlenbeck process variable that solves the SDE.

(7) $$dx_{t}=\alpha_{x}\left(\theta_{x}-x_{t}\right)dt+\sigma_{x}dW_{t}^{x}.$$

Given the exponential relationship between $\theta_{t}^{y}$ and $x_{t}$ as $\theta_{t}^{y}=\beta_{1}+\beta_{2}\exp\left(\beta_{3}x_{t}\right)$, by taking differentials with respect to *t* on both sides yields to $d\theta_{t}^{y}=\beta_{2}d\exp\left(\beta_{3}x_{t}\right)$. Let $z_{t}=\exp\left(\beta_{3}x_{t}\right)$, again by Itô’s lemma and use Equation (7), one has

$$dz_{t}=\alpha_{x}\beta_{3}\left(\theta_{x}-x_{t}+\left(\sigma_{x}^{2}\beta_{3}\right)/\left(2\alpha_{x}\right)\right)\exp\left(\beta_{3}x_{t}\right)dt+\sigma_{x}\beta_{3}\exp\left(\beta_{3}x_{t}\right)dW_{t}^{x},$$

which implies that $z_{t}$ is a geometric Ornstein–Uhlenbeck process [28]. $z_{t}$ can be expressed as

$$\log z_{t}=\log z_{0}\exp\left(-\alpha_{x}\beta_{3}t\right)+\theta_{x}\left(1-\exp\left(\alpha_{x}\beta_{3}t\right)\right)+\beta_{3}\sigma_{x}\exp\left(-\alpha_{x}\beta_{3}t\right)\int_{0}^{t}\exp\left(\alpha_{x}\beta_{3}s\right)dW_{s}^{x}.$$

Hence, $\theta_{t}^{y}=\beta_{1}+\beta_{2}\exp\left(\beta_{3}x_{t}\right)$ can be expressed as

(8) $$\begin{array}{cc} \theta_{t}^{y} & =\beta_{1}+\beta_{2}\exp\left(\beta_{3}x_{0}\exp\left(-\alpha_{x}\beta_{3}t\right)+\theta_{x}\left(1-\exp\left(-\alpha_{x}\beta_{3}t\right)\right)+\sigma_{x}\beta_{3}\exp\left(-\alpha_{x}\beta_{3}t\right)\int_{0}^{t}\exp\left(\alpha_{x}\beta_{3}s\right)dW_{s}^{x}\right). \end{array}$$

To draw a sample for trait $y_{t}$, considering the expression of $y_{t}$ in Equation (2), it suffices to recognize the dynamics of Ⓐ in Equation (3) where $Ⓐ=\int_{0}^{t}\alpha_{y}\exp\left(-\alpha_{y}\left(t-s\right)\right)\theta_{s}^{y}ds.$ By using Equation (8) for $\theta_{t}^{y}$, one has

$$Ⓐ=\beta_{1}\left(1-\exp\left(-\alpha_{y}t\right)\right)+\beta_{2}\alpha_{y}\exp\left(-\alpha_{y}t\right)A_{t}$$

where

$$A_{t}=\int_{0}^{t}\exp\left(-\alpha_{y}s+\theta_{x}+\exp\left(-\alpha_{x}\beta_{3}s\right)\left(\beta_{3}x_{0}-1+\sigma_{x}\beta_{3}\int_{0}^{s}\exp\left(\alpha_{x}\beta_{3}u\right)dW_{u}^{x}\right)\right)ds$$

is a definite integral of geometric OU process with respect to time. Currently, there is no analytical expression for $A_{t}$, so we used R package pracma [34] to draw samples of $A_{t}$ where the definite integral was computed over a finite grid by Simpson’s rule. On each grid sample of $\int_{0}^{s}\exp\left(\alpha_{x}\beta_{3}u\right)dW_{u}^{x}$ were generated by a normal variable with mean 0 and variance $\left(\exp\left(2\alpha_{x}\beta_{3}s\right)-1\right)/\left(2\alpha_{x}\beta_{3}\right)$.

**Figure 2:**
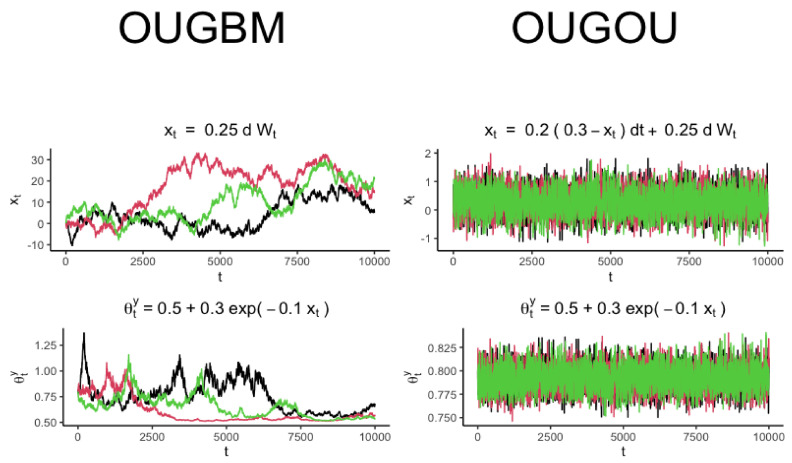
Trajectory simulation for the OUGBM and the OUGOU models. Each plot contains three realizations generated from the corresponding model. Trajectories of optimum $\theta_{t}^{y}$ were generated by evaluating the exponential relationship $\theta_{t}^{y}=0.5+0.3\exp\left(-0.1x_{t}\right)$ from the realization of covariate $x_{t}$.

Section 2.1.1 and Section 2.1.2 provide the fundamental framework for phylogenetic exponential optimal regression for adaptive trait evolution. Once Ⓐ in Equation (3) was fully recognized, samples of trait variable $y_{t}$ could be drawn accordingly by using the $y_{t}$ expressed in Equation (2). Trajectories for optimal response $\theta_{t}^{y}$ and the covariate $x_{t}$ for the OUGBM and OUGOU models are shown in Figure 2.

### 2.2. Optimal Linear Regression

Optimal $\theta_{t}^{y}$ of response trait $y_{t}$ and its covariate $x_{t}$ has a linear relationship, as follows:

(9) $$\theta_{t}^{y}=\beta_{1}+\beta_{2}x_{t}.$$

Two optimal linear regression models, OUBM [20] using a BM covariate, and OUOU [21] using an OU process covariate were developed in the literature. We included both models in this study for comparison with the optimal exponential regression model.

#### 2.2.1. OUBM Model

When the dynamic of optimum $\theta_{t}^{y}$ was assumed with a linear relationship with the BM covariate $x_{t}=\sigma_{x}W_{t}^{x}$, then

(10) $$\theta_{t}^{y}=\beta_{1}+\beta_{2}\sigma_{x}W_{t}^{x}.$$

To draw a sample for trait $y_{t}$, considering the expression of $y_{t}$ in Equation (2), it suffices to recognize the dynamics of Ⓐ in Equation (3). Ⓐ=$\int_{0}^{t}\alpha_{y}\exp\left(-\alpha_{y}\left(t-s\right)\right)\theta_{s}^{y}ds$ is computed by replacing $\theta_{t}^{y}$ with the right-hand side of Equation (10). Hence,

$$Ⓐ=\beta_{1}\left(1-\exp\left(-\alpha_{y}t\right)\right)+\beta_{2}\sigma_{x}\exp\left(-\alpha_{y}t\right)\left(W_{t}^{x}-\int_{0}^{t}\exp\left(\alpha_{y}s\right)dW_{s}^{x}\right)$$

which is a normal variable with mean $\beta_{1}\left(1-\exp\left(\alpha_{y}t\right)\right)$ and variance $\beta_{2}^{2}\sigma_{x}^{2}(t\exp\left(-2\alpha_{y}t\right)-2\left(\exp\left(-\alpha_{y}t\right)-\exp\left(-2\alpha_{y}t\right)\right)/\alpha_{y}+\left(1-\exp\left(-2\alpha_{y}t\right)\right)/\left(2\alpha_{y}\right)$.

#### 2.2.2. OUOU Model

Let $\theta_{t}^{y}=\beta_{1}+\beta_{2}x_{t}$ where $x_{t}=\theta_{x}+\exp\left(-\alpha_{x}t\right)\left(x_{0}-\theta_{x}+\sigma_{x}\int_{0}^{t}\exp\left(\alpha_{x}s\right)dW_{s}^{x}\right)$ is a random OU process variable. By replacing $x_{t}$ in terms of $t,\alpha_{x},\sigma_{x},W_{t}^{x}$ to Equation (9), one has

(11) $$\theta_{t}^{y}=\beta_{1}+\beta_{2}\theta_{x}+\beta_{2}\exp\left(-\alpha_{x}t\right)\left(x_{0}-\theta_{x}+\sigma_{x}\int_{0}^{t}\exp\left(\alpha_{x}s\right)dW_{s}^{x}\right).$$

To draw a sample for trait variable $y_{t}$, considering the expression of $y_{t}$ in Equation (2), it suffices to recognize the dynamics of Ⓐ in Equation (3), where $Ⓐ=\int_{0}^{t}\alpha_{y}\exp\left(-\alpha_{y}\left(t-s\right)\right)\theta_{s}^{y}ds$. By expressing $\theta_{t}^{y}$ in Equation (11),

Ⓐ=①+②+③

where

$$①=\int_{0}^{t}\alpha_{y}\exp\left(-\alpha_{y}\left(t-s\right)\right)\left(\beta_{1}+\beta_{2}\theta_{x}\right)ds=\left(\beta_{1}+\beta_{2}\theta_{x}\right)\left(1-\exp\left(-\alpha_{y}t\right)\right),$$

$$②=\alpha_{y}\beta_{2}\left(x_{0}-\theta_{x}\right)\int_{0}^{t}\exp\left(-\alpha_{y}t+\left(\alpha_{y}-\alpha_{x}\right)s\right)ds=-\alpha_{y}\beta_{2}\left(x_{0}-\theta_{x}\right)\left(\exp\left(-\alpha_{y}t\right)-\exp\left(-\alpha_{x}t\right)\right)/\left(\alpha_{y}-\alpha_{x}\right),$$

and

$$\begin{array}{cc} ③ & =\alpha_{y}\sigma_{x}\beta_{2}\int_{0}^{t}\exp\left(-\alpha_{y}t+\left(\alpha_{y}-\alpha_{x}\right)s\right)\int_{0}^{s}\exp\left(\alpha_{x}u\right)dW_{u}^{x}ds\\ \; & =\alpha_{y}\sigma_{x}\beta_{2}\exp\left(-\alpha_{y}t\right)/\left(\alpha_{y}-\alpha_{x}\right)\left(\exp\left(-\alpha_{x}t\right)\int_{0}^{t}\exp\left(\alpha_{x}s\right)dW_{s}^{x}-\exp\left(-\alpha_{y}t\right)\int_{0}^{t}\exp\left(\alpha_{y}s\right)dW_{s}^{x}\right) \end{array}$$

which is a normal variable with mean 0 and variance $\alpha_{y}^{2}\sigma_{x}^{2}\beta_{2}^{2}\left(\left(1-\exp\left(-2\alpha_{x}t\right)\right)/\left(2\alpha_{x}\right)+\left(1-\exp\left(-2\alpha_{y}t\right)\right)/\left(2\alpha_{y}\right)\right)/{\left(\alpha_{y}-\alpha_{x}\right)}^{2}$.

### 2.3. Optimal Adaptive-Trait Evolution along Phylogenetic Tree

A phylogenetic tree provides evidence of the summary of evolutionary history of living species [35]. For a mutation occurring in an individual identified on a lineage of the tree where the mutation changed the phenotype of the organism such as kangaroos, that mutation may change the moving style from bipedal walking to bipedal hopping. Such a mutation may need many generations to be achieved. However, the trait may be difficult to predict when a lineage is fixed for a derived trait; descendants would inherit the trait until a subsequent evolution change occurs. For a clade that contains marsupials such as kangaroos, wallabies, koalas, and possums, their differences are the results of changes after their common ancestor begins to diversify. Here, a phylogenetic tree provides information to organize this biological diversity where internal nodes depict a common ancestry and contain the formation of the degree of relatedness that is relative to the entire evolutionary history. As adopting tree thinking that living species share a common ancestor is broadly accepted in evolutionary biology, the tree provides evidence in how to conceptualize the broad sweep of biological diversity.

**Figure 3:**
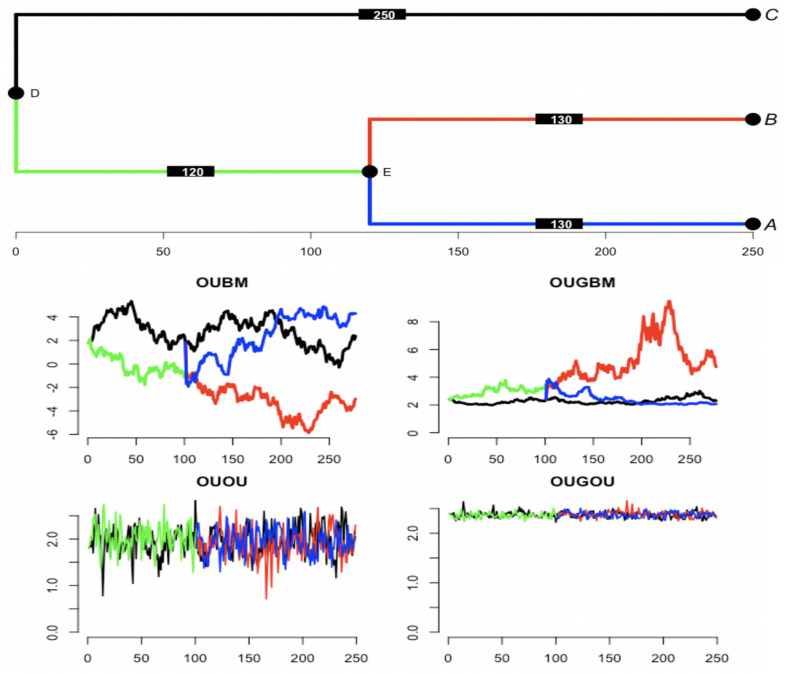
Simulation of optimal trajectories along the tree using Gaussian process covariates. (top) Tree of 3 taxa is simulated from coalescent process using R package’s ape function rcoal [36]. Original tree has branch of length 250 from root node D to tip C, 120 from node D to node E, and 130 from E to B and from E to A. Edge length is increased by multiplying a constant, and trajectories are simulated at each unit under relevant processes. Trajectories of predictor $x_{t}$ assume Brownian motion with rate $\sigma_{x}=0.5$ first simulated along the tree with $x_{0}=0$ at root ρ. For the covariate $x_{t}$ under an Ornstein–Uhlenbeck process dynamics with parameters $\alpha_{x}=0.625$, optimum $\theta_{x}=0.25$, and rate $\sigma_{x}=0.5$. Trait is first simulated along the tree with starting point $x_{0}=0$ at the root ρ. Optimum $\theta_{t}^{y}$ as a function of $x_{t}$ is computed under each model using the regression parameters $\left(\beta_{1},\beta_{2},\beta_{3}\right)=\left(1.8,0.6,-0.2\right)$. For linear model $\theta_{t}^{y}=1.8+0.6x_{t}$, and for exponential model $\theta_{t}^{y}=1.8+0.6\exp\left(-0.2x_{t}\right)$.

For trait evolution, a group of currently observed species has beautifully expressed affinity by the evolutionary tree. From the mathematical side, changes in trait value among a group of species along a phylogenetic tree can be realized by the relevant stochastic process. One realization of $y_{t}$ using a BM predictor in the OUBM and OUGBM models, and one realization of trajectories $y_{t}$ for the OU process-based predictor in the OUOU and OUGOU models using a 3-species phylogenetic tree are shown in Figure 3. Box plots of 100 simulated optimal-trait and response trait samples under the tree in Figure 3 using the tree traversal algorithm can be accessed in Figure S1 in the online Supplemental Material, displaying the spread of traits across models.

### 2.4. Approximate Bayesian Computation

Due to the exponential relationship between $\theta_{t}^{y}$ and its covariate $x_{t}$, the stochastic variable $y_{t}$ shown in Equation (2) includes a definite integral of stochastic variable $\theta_{t}^{y}$ with respect to time *t*. The distribution for the definite integral of the geometric OU process with respect to time is currently not known. Hence, the OUGOU model lack of closed-form likelihood as stochastic variable Ⓐ in Equation (3) embedded in $y_{t}$ is intractable. Approximate Bayesian computation (ABC) was used for statistical inference herein. The ABC procedure is a likelihood free based method used for model inference. To start an ABC algorithm, data are first simulated from the model using parameters drawn from prior distributions. Then, a set of the summary statistics for samples and raw data are calculated. For the ABC rejection method, a distance function d(·) and a threshold δ are used to determine posterior samples by comparing summary statistics of observed data and simulated data [37].

To determine posterior samples, we adopted the 12 summary statistics from [19], and used the mean, median, standard deviation, skewness, kurtosis, and the phylogenetic tree based statistics: the contrast mean, the contrast standard deviation, the contrast skewness, the contrast kurtosis [1], and two phylogenetics-related statistics: (i) the Bloomberg’s K statistic (measures the relatedness of species in a clade when compared to randomly selected species from the same tree T) [38] and (ii) the Pagel’s λ statistic (measures the strength of trait heritability from the ancestor) [39]. For $K=\mathrm{obs}\left(\frac{{\mathrm{MSE}}_{0}}{\mathrm{MSE}}\right)/\exp\left(\frac{{\mathrm{MSE}}_{0}}{\mathrm{MSE}}\right)$ where ${\mathrm{MSE}}_{0}$ is the mean square root of the observed tip data measured from phylogenetic correct mean and MSE is the mean squared error of the observed data calculated using the variance covariance matrix derived from the candidate tree. For trait vector Y following a Brownian motion model (i.e., $Y∼\mathrm{MVN}(\mu 1_{n},\sigma^{2}C)$), one has

$${\mathrm{MSE}}_{0}={\left(Y-\hat{\mu }1_{n}\right)}^{t}\left(Y-\hat{\mu }1_{n}\right)/\left(n-1\right)\;\mathrm{and}\;\mathrm{MSE}={\left(Z-\hat{\mu }1_{n}\right)}^{t}\left(Z-\hat{\mu }1_{n}\right)/\left(n-1\right),$$

where $\hat{\mu }=1^{t}C^{-1}Y/1^{t}C^{-1}1$ is the phylogenetic corrected mean, and Z=PY is the transformed Y vector obtained from the generalized least-square procedure. Matrix P satisfies equation DVD=I, where $V=\sigma^{2}C$ is the variance covariance matrix and I is the identity matrix. Relatively small ${\mathrm{MSE}}_{0}$ occurs when there is little covariance within the tip data that is explained by the candidate tree, and it leads to a smaller value of the ratio of ${\mathrm{MSE}}_{0}/\mathrm{MSE}$ (weaker phylogenetic signal). Conversely, while if the candidate tree precisely demonstrate the variance-covariance pattern observed in the data, then there is a small MSE, which results in large value of ${\mathrm{MSE}}_{0}/\mathrm{MSE}$ (stronger phylogenetic signal) [38]. Pagel’s λ statistic parameter λ is calculated by optimizing the likelihood function of the model, assuming that observed trait vector $Y={\left(y_{1},\cdots ,y_{n}\right)}^{t}$ follows multivariate normal distribution $Y∼MVN(\mu 1_{n},\sigma^{2}\left(\lambda C+\left(1-\lambda \right)I\right)$ where $1_{n}=\left(1,\cdots ,1\right)$ is vector of 1s, and I is an identity matrix, C is phylogentic affinity matrix transformed from the given phylogenetic tree [40]. Since both the MLE for mean μ and variance $\sigma^{2}$ can be written as a function of λ,

$$\hat{\mu }=\frac{1_{n}{\left[\lambda C+\left(1-\lambda \right)I\right]}^{-1}Y}{1_{n}^{t}{\left[\lambda C+\left(1-\lambda \right)I\right]}^{-1}1_{n}}\;\mathrm{and}\;{\hat{\sigma }}^{2}=\frac{{\left(Y-\hat{\mu }1_{n}\right)}^{t}{\left[\lambda C+\left(1-\lambda \right)I\right]}^{-1}\left(Y-\hat{\mu }1_{n}\right)}{n},$$

λ can be estimated by optimizing the likelihood function over its domain λ∈[0,1]. Those statistics resulted in a great interest in evolutionary-biology research [19,41,42]. Euclidean distance measure $d=d\left(S\left(Y,X\right),S\left(Y^{\prime },X^{\prime }\right)\right)=||S\left(Y,X\right)-S\left(Y^{\prime },X^{\prime }\right){\left|\right|}_{2}$ corresponds to those statistics *S*, where S(Y,X) and $S(Y^{\prime },X^{\prime })$ are computed from observed and simulated-trait data, respectively. The procedure for parameter estimation under the ABC rejection method is shown in Algorithm 1.
**Algorithm 1:** Approximate Bayesian computation for the models of adaptive trait evolution.**Require:** Trait datasets: response $Y={\left(y_{1},y_{2},\cdots ,y_{n}\right)}^{t}$, covariate $X={\left(x_{1},x_{2},\cdots ,x_{n}\right)}^{t}$; tree T, a threshold δ, and model $M_{j}$, starting parameter value $\Theta_{0j}$ and priors $\pi_{j}\left(\cdot \right)$ for $\Theta_{j}$, j=1,2,⋯,m.**Ensure:** Posterior samples $\Theta_{i}$, i=1,2,⋯,mLδ.1:Calculate the summary statistics $S_{0}=S\left(Y,X\right)$.2:**for**i=1,2,⋯,L**do**3:    **for** j=1,2,⋯,m **do**4:        Draw samples $\Theta_{ij}$ from prior $\pi_{j}$ under model $M_{j}$.5:        Simulate trait set $Y_{ij},X_{ij}$ under model $M_{j}$ and its parameters $\Theta_{ij}$.6:        Evaluate summary statistics $S_{ij}=S\left(Y_{ij},X_{ij}\right)$.7:        Compute distance $d_{ij}$ between $S_{0}$ and $S_{ij}$.8:    **end for**9:**end for**10:Order the distance ${\left\{d_{ij}\right\}}_{i,j=1}^{L,m}$ from the small least to the largest $\left\{d_{k},k=1,2,\cdots ,Lm\right\}$.11:**return** Posterior samples $\Theta_{i}:i=1,2,\cdots ,mL\delta$.

### 2.5. Interpretation of Change of Optimum by Its Covariate

As traits are logarithm-transformed prior to analysis, the change in response traits is measured on a ratio scale under two types of regression methods: (i) optimal linear regression or (ii) optimal exponential regression. Below, we briefly describe the change in optimum by its covariate.

(i) In optimal linear regression: First, given $\theta_{y_{1}}=\beta_{1}+\beta_{2}x_{1}$ and $\theta_{y_{2}}=\beta_{1}+\beta_{2}x_{2}$, the two equations in log scale are written as $\log\left(\theta_{y_{1}}\right)=\beta_{1}+\beta_{2}\log\left(x_{1}\right)$ and $\log\left(\theta_{y_{2}}\right)=\beta_{1}+\beta_{2}\log\left(x_{2}\right)$. The difference between the two equations is $\log\left(\theta_{y_{2}}\right)-\log\left(\theta_{y_{1}}\right)=\beta_{2}\left(\log\left(x_{2}\right)-\log\left(x_{1}\right)\right)$ which implies that $\log\left(\theta_{y_{2}}/\theta_{y_{1}}\right)=\beta_{2}\log\left(x_{2}/x_{1}\right)$. Hence, $\theta_{y_{2}}/\theta_{y_{1}}={\left(x_{2}/x_{1}\right)}^{\beta_{2}}$ depends on values of $\beta_{2}$, $x_{1}$ and $x_{2}$. Let $x_{1}=1$, $x_{2}=1.1x_{1}$ and $\beta_{2}=0.5$, then $\theta_{y_{2}}/\theta_{y_{1}}={\left(1.1x_{1}/x_{1}\right)}^{0.5}={\left(1.1\right)}^{0.5}=1.0488$, which means that a 10% increase in the covariate *x* results in 4.88% increase in the optimum of response $\theta_{y}$.

(ii) In optimal exponential regression: First, given $\theta_{y_{1}}=\beta_{1}+\beta_{2}\exp\left(\beta_{3}x_{1}\right)$ and $\theta_{y_{2}}=\beta_{1}+\beta_{2}\exp\left(\beta_{3}x_{2}\right)$, the two equations in log scale are written as $\log\left(\theta_{y_{1}}\right)=\beta_{1}+\beta_{2}\exp\left(\beta_{3}\log\left(x_{1}\right)\right)$ and $\log\left(\theta_{y_{2}}\right)=\beta_{1}+\beta_{2}\exp\left(\beta_{3}\log\left(x_{2}\right)\right)$. The difference between the two equations is $\log\left(\theta_{y_{2}}\right)-\log\left(\theta_{y_{1}}\right)=\beta_{2}x_{2}^{\beta_{3}}-\beta_{2}x_{1}^{\beta_{3}}$, which implies that $\log\left(\theta_{y_{2}}/\theta_{y_{1}}\right)=\beta_{2}\left(x_{2}^{\beta_{3}}-x_{1}^{\beta_{3}}\right)$. Hence, $\theta_{y_{2}}/\theta_{y_{1}}=\exp\left(\beta_{2}\left(x_{2}^{\beta_{3}}-x_{1}^{\beta_{3}}\right)\right)$ depends on covariate $x_{1},x_{2}$. Let $\beta_{2}=0.5,\beta_{3}=1$ and $x_{2}=1.1x_{1}$, then $\theta_{y_{2}}/\theta_{y_{1}}=\exp\left(0.5\left(1.1x_{1}-x_{1}\right)\right)$. Set $x_{1}=1$, then $\theta_{y_{2}}/\theta_{y_{1}}=\exp\left(0.5\left(\left(1.1\right)-1\right)\right)=1.0513$. So, a 10% increase in covariate *x* would result in a 5.13% increase in optimal response $\theta_{t}^{y}$.

## 3. Results

### 3.1. Simulation

#### 3.1.1. Parameter Estimation

To validate the new models, their performance was assessed through extensive simulations. Prior parameter distributions were assumed to be independent. Some appropriate priors were selected because of the models’ lack of tractable likelihood without a conjugate prior. A balanced tree of 16, 32, 64 and 128 with a height of 1, and Grafen branch length simulated by R: ape was used for the simulation. To obtain reliable estimates, 2000 (=500 × 4) posterior samples were obtained from four runs, in each run, 50,000 samples were generated, and a tolerance rate (δ=0.01) was used to obtain 500 posterior samples. Two sets of true parameters and priors were used for simulation. For the first set, all priors used uniform distribution. For the second set, priors were set to a specific distribution by intuitive beliefs about the true values of the parameters [43,44]. For the nonuniform prior, $\theta_{x}$ was assumed to be normal, as it was reasonable to assumed that the optimum remained at the peaks. An example of using the normal prior comes from a study of coral polyp evolution [19], where a suitable prior for the adaptive optimum $\theta_{x}$ of polyp thickness used the normal distribution of polyp thickness across all corals. The exponential prior was used for force parameters $\alpha_{x}$ and $\alpha_{y}$, and the inverse gamma was used for the rate parameters $\sigma_{x}$ and $\sigma_{y}$. The setup of hyperparameters for priors is listed in Table 1.

Root state $\rho =(\rho_{y},\rho_{x},\rho_{\theta })$ was set to a trivial value of 0 for all models. For each taxon size, one trait was simulated under each model from the simulation.

The results for uniform priors from this simulation of model parameters are shown in Table 2. The results for the second set using informative priors from this simulation of model parameters are shown in Tables S2 and S3 in the online Supplemental Material.

Overall, parameters could be estimated reasonably well with acceptable accuracy. The posterior mean of each parameter was close to the true parameter value under uniform priors. Results for the uniform priors from this simulation of regression parameters are shown in Table 3. On each taxon, most models showed reasonable mean estimates for $\beta_{1}$ (true 0), $\beta_{2}$ (true 1), $\beta_{3}$ (true −0.5). Results guaranteed that Algorithm 1 provided a reliable procedure for estimating parameters.

#### 3.1.2. Cross-Validation

Cross-validation is used to investigate how many taxa are needed and whether the correct model can be chosen from a candidate set. Leave-one-out cross-validation was performed under ABC using the R: abc package [37]. The balanced trees of taxon sizes 64, 128, 256, and 512 taxa were simulated using R: ape package, while 10,000 birth–death trees of taxon sizes 50, 100, 200, and 500 with birth rate 2, death rate 0.5, the time since origin 2, and probability of 0.5 for each tip were included in the final tree and simulated using the R: TreeSim package [45]. One trait datum was simulated along a given tree using parameters with values set up in Table 1 using uniform distribution. To assess if ABC could distinguish between the models, the 12 summary statistics were calculated in each model. For each model, the size of the cross-validation samples was set to 100.

**Figure 4:**
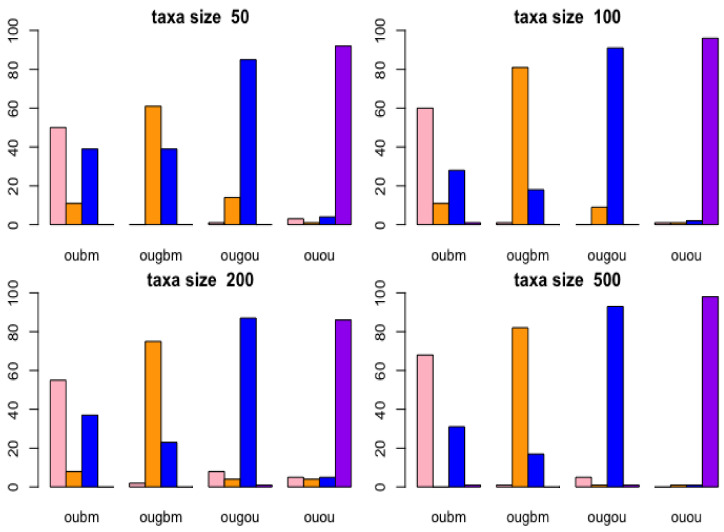
Cross-validation of models using birth-death tree. Bar plots show results of confusion matrices from cross-validation analysis under Approximate Bayesian Computatin (ABC) multinomial logistic-regression method for models of adaptive trait evolution. Four taxon sizes of 50, 100, 200, and 500 of birth-death trees were considered. The actual model is shown in the horizontal label for each bar plot on each panel, and the frequency of correctly identifying the models is represented by the height of the bar plots.

Results of the confusion matrix are reported with birth-death tree cases by bar plots in Figure 4. In the lower right panel (taxon size 500) in Figure 4, the bar plots in the OUGOU categories shows that for ABC model choice will identify the OUOU model 1 time, the OUGOU model 93 times, the OUGBM model 1 time, the OUBM model 5 times, which yielded to the misclassification proportion for the OUGOU model of (1 − 93/100) × 100% = 7%; in the upper left panel (taxon size 50), the rightmost bar plots in the OUOU categories shows that the ABC model identified the OUOU model (purple) 90 times, the OUGOU model (blue) 4 times, the OUGBM model (orange) 1 time and the OUBM model (pink) 3 times among the 100 samples, which yielded to the misclassification proportion for the OUOU model of (1 − 90/100) × 100% = 10%.

From this analysis, models are distinguishable at each taxon size. When taxa increase, ABC can more frequently identify the correct models. There are other factors, such as the choices of parameters and number of models in the candidates set, which may impact the power of correctly identifying the correct models. Here, we used constant factors. Results of the confusion matrix for each model reported with the balanced tree cases were similar to those of the case with the birth-death tree cases, and can be accessed in Figure S2 in the online Supplemental Material.

### 3.2. Empirical Analysis

**Figure 5:**
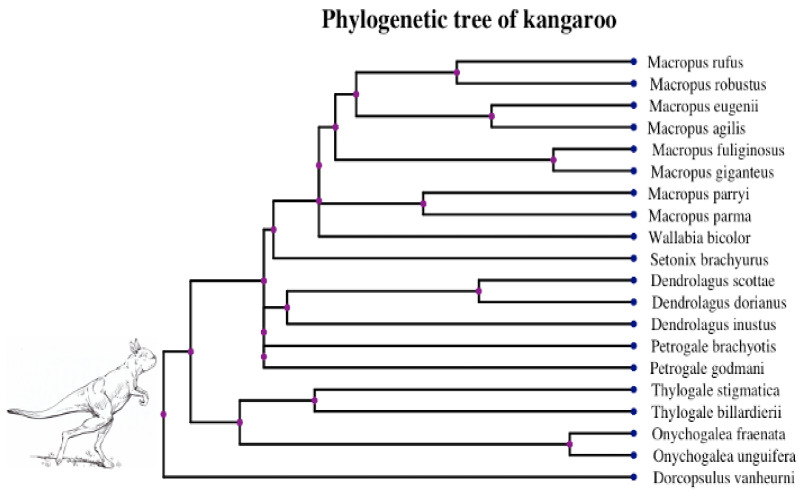
Phylogenetic tree of 20 giant kangaroo species in [18]. Image at the root of the tree is a reconstruction of *Sthenurus stirlingi* [47], an extinct giant kangaroo in walking pose.

Kangaroos are bipedal, and using their femoral midshaft circumference is especially suitable for predicting body mass. We used the trait datasets in [18] and applied our models by treating femoral bone circumference as the covariate to explore its impact on the optimum of body mass. The phylogeny of kangaroos is shown in Figure 5 and trait values corresponding to the species can be accessed in Table S3 in the Supplemental Material. Prior to log transformation, data were scaled by the feature-scaling method [46], while the curved relationship remains unchanged under this scaling. Our ABC algorithm worked properly for the dataset where traits were simulated within a reasonable range.

Posterior means for the parameters of each model is shown in Table 4.

For the kangaroo dataset, all models reported relatively small estimates of force parameters $\alpha_{x}$ and $\alpha_{y}$, which indicated that relative weak force was detected to pull the trait back to its optimum during evolution. For rate parameter, $\sigma_{x}$, the OU*OU models reported a smaller value than that of OU*BM models, while $\sigma_{y}$ for the response trait in all model was between 0.5 and 0.8. For optimum parameter for the covariate trait, $\theta_{x}$, both exponential model (OUGOU) and linear model reported negative values. For regression parameters, linear models (OUBM and OUOU) reported a positive regression slope $\beta_{2}$ which was consistent with [18], where positive correlation among traits was reported. Regression parameters $\beta_{i},i=1,2,3$ reported relatively closed values across the same class of models. Overall, our results predicted that bone circumference has a positive effect on the optimum body mass, which is consistent with the result in [18] when using phylogenetic independent contrast as the response trait.

We used Bayes factors(BF) to compare the models. The posterior probability P(M|D) of a model M given data D is given by Bayes’ theorem: Pr(M|D)=Pr(D|M)Pr(M)/Pr(D). We adopted the method in [19] and computed the BF, defined as the ratio of the posterior model probabilities of two different models $M_{i}$ and $M_{j}$, parameterized by model parameter vectors $\Theta_{i}$ and $\Theta_{j}$. This is performed by using function postpr in the R package abc [37], where posterior model probabilities are estimated using the rejection method.

The model comparison under the Bayes factor is shown in Table 5. For the kangaroo data in [18], the best model was the OUGBM model, followed by the OUGOU, OUBM, and OUOU models. Their pairwise Bayes factors are shown in Table 5. The best model (Rank = 1st) was the OUGBM model. This dataset provides relative equal support for all the exponential OUG** models, a result which was slightly higher than the linear OU** models with the Bayes factor 1.5000 for OUGBM model over OUBM, and 2.1132 for OUGBM over OUOU model. This indicates that the evolution of the optimum $\theta_{t}^{y}$ was also more appropriately described by the geometric BM process predictor than that described by a linear predictor.

**Figure 6:**
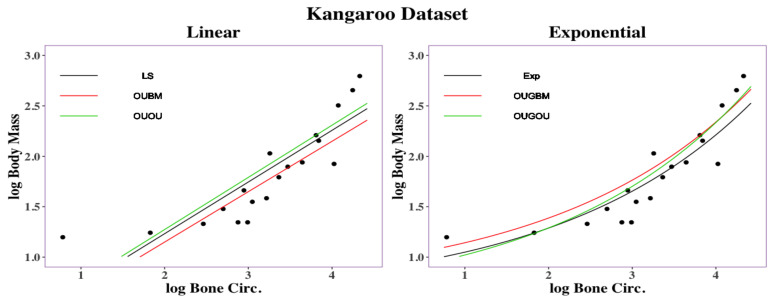
Regression curves for kangaroo traits: femoral bone circumference vs. body mass. Linear regression had an RMSD =0.2748 under the LS method, RMSD =0.2924 under the OUBM model, and RMSD =0.2788 under the OUOU model. Exponential regression had an RMSD =0.2122 under the exponential method, RMSD =0.2228 under the OUGBM model, and RMSD =0.1982 under the OUGOU model.

Regression curves are shown in Figure 6. Overall, the exponential models (EXP, OUGBM, and OUGOU) returned smaller RMSD values than those of the linear models (LS, OUBM, and OUOU) suggesting the utility of the new models. To interpret the impact on the optimal $\theta_{y}$ by its covariate *x*, we again used the two transformation methods described in Section 2.5 and the posterior mean of parameters in Table 4.

For a 1% decrement of the covariate bone circumference across kangaroos, it was expected that there would be a 0.13% decrement of the body mass $\theta_{y}$ under the EXP model, 0.13% decrement under the OUGBM model, 0.14% decrement under the OUGOU model, 0.51% decrement under the LS model, 0.50% decrement under the OUBM model and 0.52% decrement under the OUOU model. For a 5% increment of the covariate bone circumference across the kangaroos, it was expected that there would be a 0.62% increment of the body mass $\theta_{y}$ under the EXP model, 0.63% increment under the OUGBM model, 0.69% decrement under the OUGOU model, 2.53% increment under the LS model, 2.47% decrement under the OUBM model, and 2.56% increment under the OUOU model. Overall, the exponential models predicted smaller optimum changes of the optima than the linear models did for this dataset. A list of optimum changes corresponding to the covariate under those models can be seen in Table S4 in online Supplemental Material.

## 4. Discussion

Two phylogenetic optimal exponential regression models, OUGBM and OUGOU, for adaptive trait evolution under stabilizing selection were developed. Simulations showed that the new models were validated where posterior means of parameters were close to their true parameter values. The utility of the new regression models in phylogenetic comparative analysis is accessed by analyzing the kangaroo dataset, and results showed that the new models could be appropriately used and are more competitive than the linear models.

Parameter estimation for regression parameters in the ABC procedure depends on several factors. While appropriate priors are required for simulating samples, the choice of the hyperparameters is also important. In this study, uniform distribution with bounds of regression estimates ±5 times their standard deviations was used. As results showed the fit of the model, the choice of the parameters for ABC inference provides a reasonable range to cover the true parameters.

The OU process is applied to model stabilizing selection, but is currently criticised for simply being a trait-tracking movement process [48]. Our models assumed that the optimum was tracked by its covariates in a nonlinear functional manner. While our approach provides options for analyzing trait data from the aspect of adaptive trait evolution, it remains to be seen whether models can accurately estimate the adaptive optima from the stabilizing selection, as described in the literature [48]. Undoubtedly, it would be very interesting to investigate this open question for all OU process-based PCMs [8].

Phylogenetic comparative methods are very useful statistical methods to answer evolutionary questions. Those methods, which were developed on the basis of the property of stochastic process remains, require more improvement so that they are able to face the challenges of an intrinsic evolutionary process, which merely a simple Brownian motion model or an OU process model can solve [40,49]. Our models provide feasible options to users in the community to account for nonlinearity in the relationship between the trait optima undergoing stabilizing selection and predictor traits. The models and procedures included in this study were implemented into the R package ouxy [50].

## Figures and Tables

**Table 1:** Simulation setup for true parameter values and prior distributions. U, uniform distribution; E, exponential distribution; IG inverse gamma distribution; and N, normal distribution. In inverse gamma distribution, sh = shape and sc = scale.

Par	True 1	Prior 1	True 2	Prior 2
$\alpha_{y}$	0.50	U(0,1)	0.20	E (rate = 5)
$\alpha_{x}$	0.125	U(0,0.25)	0.125	E (rate = 8)
$\theta_{x}$	0.00	U(−5,5)	1.00	N(mean = 1, sd = 1)
$\sigma_{x}$	2.50	U(0,5)	0.5	IG (sh = 2,sc = 0.5)
$\sigma_{y}$	1.00	U(0,2)	0.5	IG (sh = 2, sc = 0.5)
$\beta_{1}$	0.00	U(−1,1)	0.00	U(−5,5)
$\beta_{2}$	1.00	U(0,2)	−2.00	U(−7,3)
$\beta_{3}$	−0.50	U(−1,0)	−0.5	U(2,−3)

**Table 2:** Simulation results of validating models through model parameter estimation using uniform prior. Four different taxon sizes of 16, 32, 64, and 128 were used for the four models (OUGBM, OUGOU, OUBM and OUOU). Means and 95% credible intervals using 2000 posterior samples from 4 individual runs on each model are reported for each model parameter on each column.

Model	Taxa	$\;\;\;\;\alpha_{y}$	$\;\;\;\;\alpha_{x}$	$\;\;\;\;\theta_{x}$	$\;\;\;\;\sigma_{x}$	$\;\;\;\;\sigma_{y}$
	**True Value**	0.5	0.125	0	2.5	1
OUGBM	16	0.52 (0.06, 0.96)			2.16 (0.26, 4.59)	0.89 (0.16, 1.83)
	32	0.53 (0.08, 0.95)			1.83 (0.25, 4.3)	0.95 (0.2, 1.82)
	64	0.54 (0.09, 0.95)			1.66 (0.2, 4.1)	0.93 (0.2, 1.78)
	128	0.52 (0.08, 0.95)			1.65 (0.21, 4.07)	0.91 (0.2, 1.78)
OUGOU	16	0.44 (0.04, 0.95)	0.12 (0.01, 0.24)	−1.14 (−4.49, 2.81)	2.25 (0.65, 4.28)	1.16 (0.38, 1.88)
	32	0.47 (0.04, 0.95)	0.12 (0.01, 0.24)	−1.22 (−4.59, 2.75)	2.52 (0.82, 4.56)	0.99 (0.19, 1.83)
	64	0.48 (0.04, 0.95)	0.12 (0.01, 0.24)	−1.16 (−4.58, 2.93)	2.61 (0.87, 4.59)	0.95 (0.18, 1.81)
	128	0.49 (0.04, 0.95)	0.12 (0.01, 0.24)	−1.16 (−4.58, 2.88)	2.57 (0.79, 4.57)	0.9 (0.16, 1.78)
OUBM	16	0.5 (0.05, 0.95)			2.14 (0.59, 4.22)	1.13 (0.11, 1.92)
	32	0.56 (0.07, 0.96)			2.05 (0.55, 4.21)	1.05 (0.1, 1.92)
	64	0.52 (0.06, 0.96)			1.95 (0.48, 4.12)	1.07 (0.11, 1.92)
	128	0.54 (0.06, 0.96)			1.92 (0.51, 4.05)	1.06 (0.11, 1.91)
OUOU	16	0.53 (0.05, 0.95)	0.12 (0.01, 0.24)	0.63 (−4.15, 4.49)	2.13 (0.64, 4.11)	1.08 (0.12, 1.92)
	32	0.55 (0.05, 0.95)	0.12 (0.01, 0.24)	0.94 (−4.15, 4.56)	1.9 (0.42, 4)	1.06 (0.13, 1.9)
	64	0.53 (0.05, 0.94)	0.12 (0.01, 0.24)	0.79 (−4.18, 4.54)	1.85 (0.42, 3.96)	1.06 (0.12, 1.91)
	128	0.55 (0.05, 0.95)	0.12 (0.01, 0.24)	0.79 (−4.26, 4.54)	1.81 (0.44, 3.92)	1.05 (0.11, 1.9)

**Table 3:** Simulation results of validating models through regression parameter estimation using uniform prior. Four different taxon sizes of 16, 32, 64, and 128 were used for four models (OUGBM, OUGOU, OUBM and OUOU). Means and 95% credible intervals using 2000 posterior samples from 4 individual runs on each model were reported for each regression parameter on each column.

Model	Taxa	$\;\;\;\;\;\;\beta_{1}$	$\;\;\;\;\;\;\beta_{2}$	$\;\;\;\;\;\;\beta_{3}$
	**True Value**	0	1	−0.5
OUGBM	16	−0.08 (−0.91, 0.84)	1.01 (0.14, 1.89)	−0.46 (−0.94, −0.05)
	32	−0.02 (−0.9, 0.87)	0.95 (0.12, 1.87)	−0.47 (−0.95, −0.05)
	64	−0.02 (−0.91, 0.89)	0.96 (0.14, 1.86)	−0.48 (−0.95, −0.05)
	128	0.01 (−0.9, 0.9)	0.97 (0.14, 1.86)	−0.48 (−0.95, −0.04)
OUGOU	16	−0.01 (−0.92, 0.88)	0.88 (0.06, 1.89)	−0.47 (−0.92, −0.05)
	32	−0.03 (−0.92, 0.88)	0.89 (0.07, 1.89)	−0.48 (−0.94, −0.05)
	64	−0.05 (−0.92, 0.88)	0.88 (0.07, 1.89)	−0.48 (−0.93, −0.05)
	128	−0.05 (−0.92, 0.88)	0.91 (0.07, 1.89)	−0.49 (−0.94, −0.05)
OUBM	16	−0.03 (−0.88, 0.89)	0.8 (0.11, 1.81)	
	32	0.01 (−0.89, 0.9)	0.78 (0.09, 1.82)	
	64	−0.01 (−0.9, 0.89)	0.79 (0.09, 1.83)	
	128	−0.02 (−0.9, 0.89)	0.8 (0.09, 1.83)	
OUOU	16	−0.11 (−0.9, 0.88)	0.86 (0.11, 1.81)	
	32	−0.11 (−0.9, 0.88)	0.81 (0.09, 1.83)	
	64	−0.1 (−0.89, 0.88)	0.85 (0.1, 1.85)	
	128	−0.09 (−0.89, 0.88)	0.82 (0.1, 1.84)	

**Table 4:** Posterior means of parameters for kangaroo dataset.

Model	Parameter							
	$\alpha_{x}$	$\alpha_{y}$	$\sigma_{x}$	$\sigma_{y}$	$\theta_{x}$	$\beta_{1}$	$\beta_{2}$	$\beta_{3}$
EXP						0.5987	0.2946	0.4251
OUGBM		0.0016	1.3420	0.7888		0.6848	0.2985	0.4281
OUGOU	0.0014	0.0015	0.8034	0.5480	−1.2113	0.5208	0.3258	0.4293
LS						0.2078	0.5125	
OUBM		0.0015	1.4413	0.7392		0.1504	0.4996	
OUOU	0.0014	0.0014	0.9952	0.6931	−0.5732	0.2392	0.5713	

**Table 5:** Bayes factor table for kangaroo dataset. Posterior probability P(M|D) for each model is shown in the first row; and models shown in second row. Bayes factor ${\mathrm{BF}}_{ij}$ for model $M_{i}$ vs. model $M_{j}$ shown in *i*th row and *j*th column. Acceptance rate was set to 1% (δ=0.01) for the kangaroo dataset.

	P(M|D)	0.3360	0.2810	0.2240	0.1590
**Rank**	**Model** M	**OUGBM**	**OUGOU**	**OUBM**	**OUOU**
1st	OUGBM	1.0000	1.1957	1.5000	2.1132
2nd	OUGOU	0.8363	1.0000	1.2545	1.7673
3rd	OUBM	0.6667	0.7972	1.0000	1.4088
4th	OUOU	0.4732	0.5658	0.7098	1.0000

## Data Availability

The data presented in this study are available on request from the corresponding author.

## Supplementary Materials

The following are available at https://www.mdpi.com/1099-4300/23/2/218/s1, Figure S1: Box plots of simulated trait values, Figure S2: Cross-validation of models using balanced tree, Table S1: Simulation results of validating models through model parameter estimation using informative priors, Table S2: Simulation results of validating models through regression parameter estimation using informative priors, Table S3: Body mass and bone circumference for Kangaroo species, Table S4: Percentage change of the optimal trait impacted by its covariate.
