# Supplementary material for: Phylogenetic Curved Optimal Regression for Adaptive Trait Evolution
Source: Entropy (Basel). 2021 Feb 10;23(2):218. doi: 10.3390/e23020218 (PMC7916804; doi:10.3390/e23020218)
Supplement: Supplementary file 1 [file entropy-23-00218-s001.pdf]

Supplemental Materials: Phylogenetic Curved Optimal Regression for Adaptive Trait Evolution

Dwueng-Chwuan Jhwueng*

Department of Statistics, Feng-Chia University
No 100, Seatwen, Taichung Taiwan

Chih-Ping Wang

Department of Statistics, Feng-Chia University
No 100, Seatwen, Taichung Taiwan

---

*Tel.:011-886-4-24517250 ext 4402
Email address: dcjhwueng@fcu.edu.tw (Dwueng-Chwuan Jhwueng)

1. Box Plots of Trait and Optimum

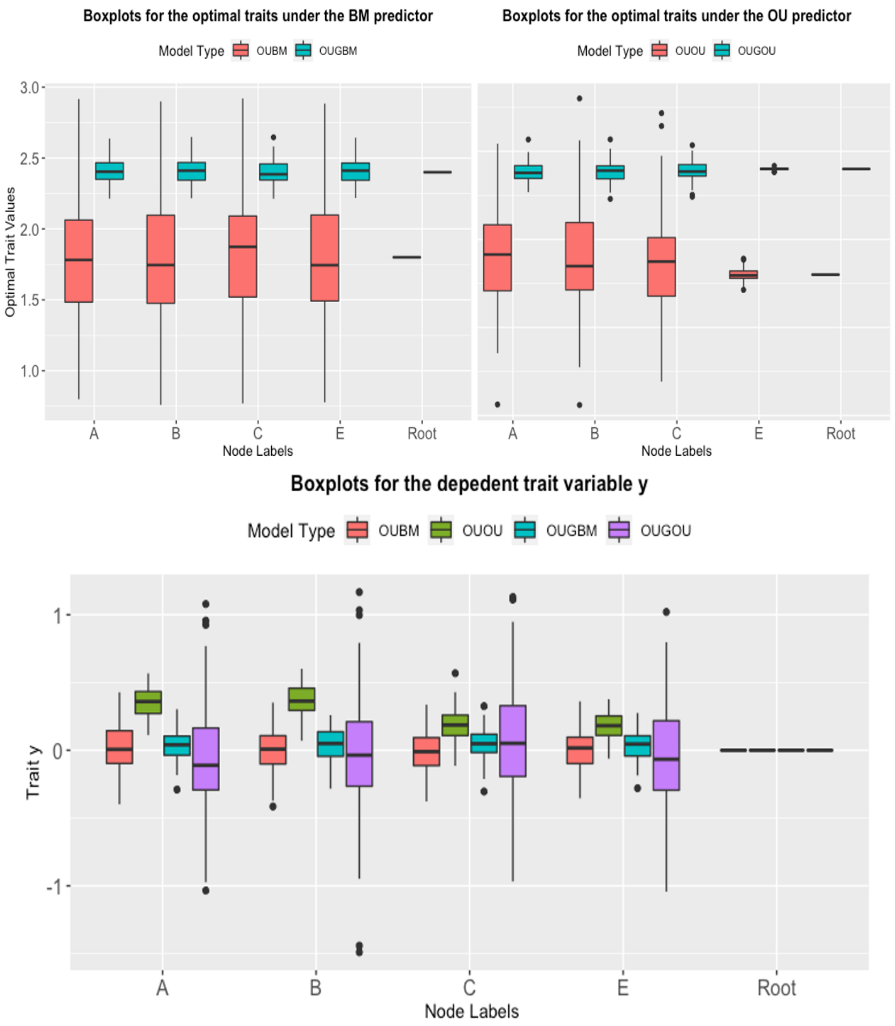

Figure S1: Box plots of simulated trait values. (top left), optimum θ_t^y is assumed with exponential relationship with a Brownian motion predictor x_t; (top right) optimal θ_t^y is assumed with exponential relationship with an Ornstein-Uhlenbeck predictor x_t. (bottom) Response traits y_t simulated at each nodes. The root of the tree were assumed with fixed value for each model type. The root for the predictor $x_0 = 0$. For the OUBM and OUOU models, the root is $\theta_0^y = 1.8$; for the OUGBM and OUGOU models, $\theta_0^y = 1.8 + 0.6 = 2.4$.True parameters $\alpha_x = 0.15, \alpha_y = 0.2, \sigma_x = 0.1, \sigma_y = 0.15, \theta_x = 0, \beta_1 = 0.1, \beta_2 = 1$ and $\beta_3 = -0.2$. Trait at the nodes were simulated under each models where each boxplot was generated by 100 replicates.

2. Simulation Results

2.1. Informative Prior

2.1.1. Tables

Table S1: Simulation results of validating models through model parameter estimation using informative priors. Four different taxon sizes of 16, 32, 64, and 128 were used for the four models (OUGBM, OUGOU, OUBM and OUOU). Means and 95% credible intervals using 2000 posterior samples from 4 individual runs on each model are reported for each model parameter on each column.

Model	Taxa	α_y	α_x	θ_x	σ_x	σ_y
	**True Value**	**0.5**	**0.125**	**1**	**0.5**	**0.5**
OUGBM	16	0.28 (0.03,1.27)			0.25 (0.11,0.55)	0.27 (0.11,0.65)
	32	0.25 (0.03,1.2)			0.25 (0.11,0.54)	0.27 (0.12,0.6)
	64	0.25 (0.02,1.12)			0.25 (0.11,0.53)	0.28 (0.12,0.6)
	128	0.24 (0.02,1.12)			0.26 (0.11,0.55)	0.3 (0.13,0.65)
OUGOU	16	0.23 (0.02,1.07)	0.1 (0.01,0.37)	−0.26 (−1.87,1.4)	0.26 (0.11,0.54)	0.31 (0.13,0.69)
	32	0.24 (0.02,1.06)	0.1 (0.01,0.38)	−0.29 (−1.8,1.35)	0.29 (0.13,0.57)	0.31 (0.13,0.66)
	64	0.24 (0.02,1.06)	0.1 (0.01,0.39)	−0.21 (−1.73,1.35)	0.29 (0.12,0.58)	0.32 (0.14,0.66)
	128	0.24 (0.02,1.05)	0.09 (0.01,0.38)	−0.16 (−1.66,1.36)	0.29 (0.13,0.59)	0.34 (0.14,0.67)
OUBM	16	0.29 (0.02,1.09)			0.32 (0.16,0.62)	0.3 (0.11,1.17)
	32	0.3 (0.02,1.22)			0.31 (0.15,0.61)	0.29 (0.11,1.12)
	64	0.3 (0.02,1.24)			0.3 (0.15,0.59)	0.3 (0.11,1.13)
	128	0.29 (0.02,1.23)			0.31 (0.15,0.61)	0.3 (0.11,1.13)
OUOU	16	0.33 (0.02,1.29)	0.07 (0,0.33)	0.14 (−1.39,1.76)	0.33 (0.14,0.68)	0.31 (0.11,1.27)
	32	0.31 (0.02,1.29)	0.07 (0,0.34)	0.25 (−1.34,1.84)	0.35 (0.15,0.69)	0.3 (0.1,1.27)
	64	0.31 (0.02,1.31)	0.08 (0.01,0.34)	0.27 (−1.33,1.84)	0.34 (0.15,0.67)	0.31 (0.11,1.27)
	128	0.31 (0.02,1.27)	0.08 (0,0.34)	0.28 (−1.34,1.85)	0.34 (0.15,0.67)	0.31 (0.11,1.27)

Table S2: Simulation results of validating models through regression parameter estimation using informative priors. Four different taxon sizes of 16, 32, 64, and 128 were used for four models (OUGBM, OUGOU, OUBM and OUOU). Means and 95% credible intervals using 2000 posterior samples from 4 individual runs on each model were reported for each regression parameter on each column.

**Model**	**Taxa**	$\boldsymbol{\beta_1}$	$\boldsymbol{\beta_2}$	$\boldsymbol{\beta_3}$
	**True Value**	**0**	**2**	**− 0.5**
OUGBM	16	−0.14 (−4.61,4.54)	−1.53 (−6.24,2.01)	−0.18 (−2.65,1.77)
	32	−0.32 (−4.61,4.39)	−1.86 (−6.24,1.72)	−0.37 (−2.7,1.73)
	64	−0.28 (−4.55,4.39)	−1.98 (−6.3,1.72)	−0.4 (−2.75,1.73)
	128	−0.18 (−4.52,4.4)	−1.99 (−6.39,1.72)	−0.42 (−2.71,1.73)
OUGOU	16	−0.72 (−4.56,4)	−2.07 (−6.53,2.41)	−0.38 (−2.64,1.82)
	32	−0.74 (−4.48,4.16)	−2.09 (−6.54,2.39)	−0.45 (−2.66,1.8)
	64	−0.71 (−4.44,4.08)	−2.12 (−6.52,2.41)	−0.42 (−2.7,1.78)
	128	−0.67 (−4.42,4.1)	−2.13 (−6.53,2.41)	−0.42 (−2.71,1.76)
OUBM	16	0.72 (−4.03,4.35)	−1.75 (−6.04,2.49)	
	32	0.86 (−4.03,4.41)	−1.33 (−5.86,2.56)	
	64	0.64 (−4.1,4.33)	−1.55 (−6.05,2.58)	
	128	0.56 (−4.12,4.34)	−1.34 (−5.92,2.6)	
OUOU	16	−0.32 (−4.22,4.31)	−1.21 (−6.13,2.61)	
	32	−0.27 (−4.32,4.2)	−1.26 (−6.04,2.6)	
	64	−0.15 (−4.31,4.3)	−1.18 (−6.04,2.58)	
	128	0.05 (−4.27,4.35)	−1.3 (−6.1,2.58)	

3. Cross-Validation

3.1. Cross-Validation Result using Balanced Tree

Results of the confusion matrix by bar plot for each model are reported with balanced tree cases in Figure S2. In the upper left panel (taxon size 64), the bar plots in the OUGBM category shows that for ABC model choice will identify the OUBM model (pink) 1 times, the OUGBM model (orange) 69 times, the OUGOU model (blue) 0 times, and OUOU model (purple) 0 times, which yielded to the misclassification proportion for the OUGBM model of (1-69/100) x 100% = 31%; in the lower right panel (taxon size 512), the bar plots in the OUGOU categories the ABC model identified the OUBM model (pink) 0 times, the OUGBM model (orange) 6 times, the OUGOU model (blue) 94 times and the OUOU model (purple) 0 times, which yielded to the misclassification proportion for the OUGOU model of (1-94/100) x 100% = 6%.

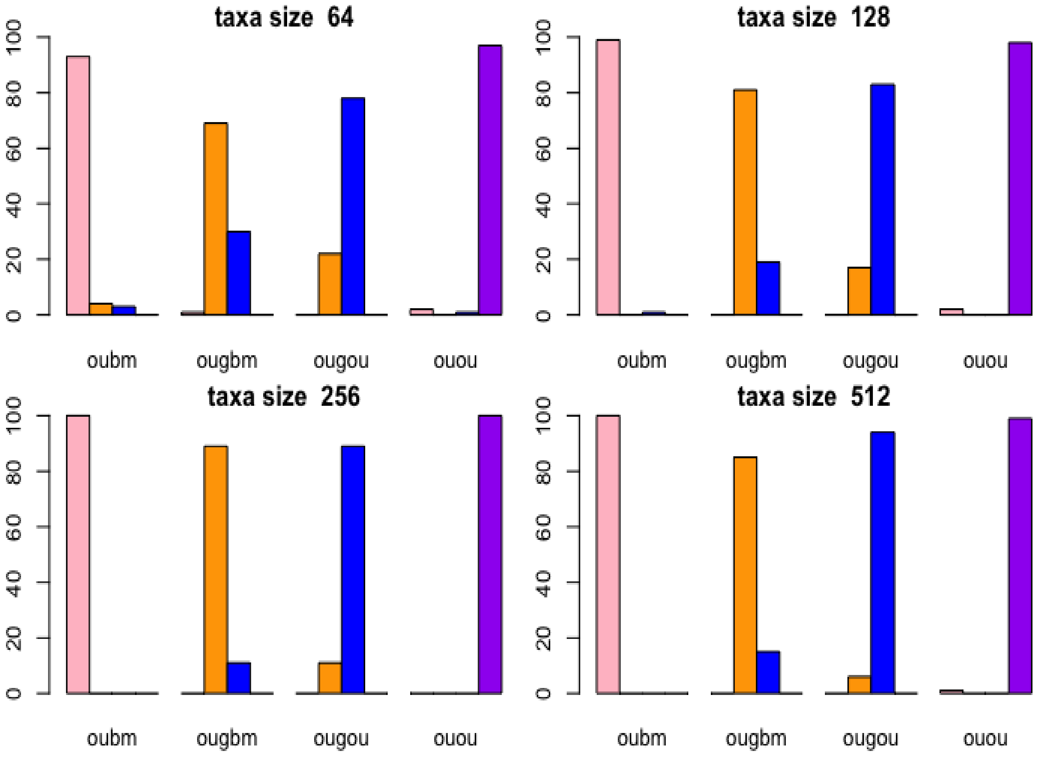

Figure S2: Cross-validation of models using balanced tree. Bar plots show results of confusion matrices from cross-validation analysis under Approximate Bayesian Computation (ABC) multinomial logistic-regression method for models of adaptive trait evolution. Four taxon sizes of 64, 128, 256, and 512 of balanced were considered. The actual model is shown in the horizontal label for each bar plot on each panel, and the frequency of correctly identifying the models is represented by the height of the bar plots.

4. Empirical Data

4.1. Kangaroo Traits

Trait values of kangaroos are shown in Table S3.

Table S3: Body mass and bone circumference for 26 Kangaroo species [1] corresponding to the phylogenetic relationship in the manuscript. $\bar{C}$: mean circumference (mm), $sd(C)$: standard deviation of circumference (mm), $\bar{M}$: mean body mass (kg), $sd(M)$: standard deviation of body mass (kg), n: number of samples.

	**Species**	$\bar{C}$	$sd(C)$	$\bar{M}$	$sd(M)$	n
1	Dendrolagus dorianus	39.75	0.35	8.75	0.35	2
2	Dendrolagus inustus	51.00		17.00		1
3	Dendrolagus mbaiso	38.10		9.00		1
4	Dendrolagus scottae	42.00	1.41	10.50	1.41	2
5	Dorcopis muelleri	42.00	7.07	9.45	5.02	2
6	Dorcopsis atrata	40.00		7.50		1
7	Dorcopsis luctuosa	33.20		4.10		1
8	Dorcopsulus vanheurni	24.00		2.00		1
9	Macropus agilis	58.50		11.00		1
10	Macropus eugenii	32.90		6.80		1
11	Macropus fuliginosus	77.33	10.97	46.33	29.67	3
12	Macropus giganteus	68.17	9.11	30.60	11.66	35
13	Macropus parma	27.82	3.53	2.85	0.67	5
14	Macropus parryi	52.00		15.70		1
15	Macropus robustus	60.50		25.30		1
16	Macropus rufogriseus	47.08	4.83	11.98	3.35	4
17	Macropus rufus	72.42	8.03	36.28	13.99	32
18	Onychogalea fraenata	33.50		3.00		1
19	Onychogalea unguifera	37.00		5.75		1
20	Petrogale brachyotis	34.30		5.30		1
21	Petrogale godmani	37.70		13.00		1
22	Petrogale mareeba	29.50		2.56		1
23	Setonix brachyurus	32.00		3.00		1
24	Thylogale billardierii	21.20		1.60		1
25	Thylogale stigmatica	30.00		4.45		1
26	Wallabia bicolor	46.24	12.47	11.28	6.62	5

5. Percentage Change of the Optimal Trait Impacted by Its Covariate

5.1. Kangaroo Dataset

Table S4: Percentage change of the optimal trait impacted by its covariate for the models for the kangaroo dataset in [1]. Each row represents the percentage change of the optimal trait in the model corresponding to the percentage change in the covariate.

Model	Covariate Change							
	-10%	-5%	-3%	-1%	1%	3%	5%	10%
EXP	-1.28	-0.63	-0.38	-0.13	0.12	0.37	0.62	1.23
OUGBM	-1.27	-0.63	-0.37	-0.12	0.12	0.37	0.61	1.21
OUGOU	-1.27	-0.63	-0.37	-0.12	0.12	0.37	0.61	1.21
LS	-5.26	-2.59	-1.55	-0.51	0.51	1.53	2.53	5.01
OUBM	-5.08	-2.51	-1.50	-0.50	0.49	1.47	2.45	4.83
OUOU	-5.04	-2.49	-1.48	-0.49	0.49	1.46	2.42	4.79

References

[1] K. M. Helgen, R. T. Wells, B. P. Kear, W. R. Gerdtz, T. F. Flannery, Ecological and evolutionary significance of sizes of giant extinct kangaroos, Australian Journal of Zoology 54 (4) (2006) 293–303.
